# Large-Scale Data Analytics of the Romanian National Inpatient Database: Prevalence, Incidence, and Mortality of Chronic Wounds, 2017–2022

**DOI:** 10.3390/medicina62030468

**Published:** 2026-02-28

**Authors:** Mona Taroi (Yassin Cataniciu), Liliana Vecerzan (Novac), Ilie Gligorea, Sorin Radu Fleacă, Doru Florian Cornel Moga, Adrian Gheorghe Boicean, Cosmin Ioan Mohor, Adrian Nicolae Cristian, Horațiu Paul Domnariu, Augusta Rațiu, Florin Daniel Sofonea, Carmen Daniela Domnariu

**Affiliations:** 1 Faculty of Medicine, Lucian Blaga University of Sibiu, 550169 Sibiu, Romania; mona.yassincataniciu@ulbsibiu.ro (M.T.); horatiupaul.domnariu@ulbsibiu.ro (H.P.D.); carmen.domnariu@ulbsibiu.ro (C.D.D.); 2 Faculty of Military Management, Nicolae Bălcescu Land Forces Academy, 550170 Sibiu, Romania; iliegligorea@gmail.com; 3 Faculty of Science, Lucian Blaga University of Sibiu, 550012 Sibiu, Romania

**Keywords:** chronic wounds, prevalence, incidence, mortality, data analytics, health informatics, computational epidemiology

## Abstract

*Background and Objectives*: Assessing the national burden of chronic wounds is a complex data analytics challenge. Robust estimates in Eastern Europe are scarce, highlighting the need for computational methods to validate cases in large-scale health databases. *Materials and Methods*: We applied a large-scale data analytics approach to Romania’s National Inpatient Database (public hospitals, 2017–2022). A computational case-ascertainment algorithm (validated “≥2 admissions” rule) was used to identify recurrently hospitalized patients, establishing a cohort of 18,856 patients (65,771 hospitalizations). We computed annual prevalence, incidence, and mortality per 100,000 adults, stratified by ulcer categories, age, and sex. *Results*: Hospital-treated prevalence and incidence showed a clear pre-pandemic peak followed by a marked decline in 2020–2021 and only partial rebound by 2022, consistent with pandemic-related disruption of inpatient care. Population-level mortality remained low, but pressure ulcers, although least frequent, accounted for the highest mortality burden. Venous ulcers were the most common category, and the hospital-treated burden was concentrated in adults aged ≥ 65 years and in men. *Conclusions*: This nationwide data-analytics framework provides the first validated inpatient indicators of chronic ulcer burden in Romania and demonstrates substantial hospital-treated disease burden with pronounced sensitivity to healthcare access constraints. *Clinical Implications:* The findings can support health-policy and prevention strategies by prioritizing early detection and integrated hospital–community wound care pathways for high-risk groups (men and older adults) and by strengthening outpatient services to reduce avoidable admissions.

## 1. Introduction

Chronic wounds, including diabetic foot ulcers, venous leg ulcers, arterial ulcers, and pressure ulcers, represent a growing public health concern worldwide [1,2,3]. These wounds are defined as skin lesions with loss of substance that do not heal within a typical timeframe and fail to respond adequately to the usual reparative mechanisms of the skin. Restoring the anatomical and functional integrity of the skin is a slow process that may take months or even years to heal [4,5]. Chronic ulcers are often complicated by infection and frequently coexist with cardiovascular disease. Together, these factors increase mortality risk and necessitate individualized wound management alongside cardiovascular optimization, often resulting in prolonged and repeated hospitalizations [6,7].

Consequently, chronic ulcers substantially increase morbidity and frailty and impose a high burden on patients, caregivers, and health systems. Large real-world costing analyses have quantified the major direct healthcare expenditure attributable to wound management [8], while prospective clinical studies in older adults with leg ulcers document high frailty prevalence and marked impairment in wound-related quality of life [9]. Population-based analyses further suggest excess mortality among patients with chronic venous disease and venous leg ulceration, underscoring the severity of advanced ulcer phenotypes captured in healthcare datasets [10].

Understanding the epidemiology and contributing factors of chronic wounds is essential for designing effective care and prevention strategies across system-level interventions and behavioural/family-level measures, including lifestyle and diet to reduce obesity, alcohol consumption, and other modifiable risk [11,12,13]. This underscores the need for ongoing, comprehensive research. While several studies have addressed the epidemiology, risk factors, and economic burden associated with chronic wounds, meaningful comparison and extrapolation of findings across different populations remain difficult [14,15,16,17].

There is considerable heterogeneity in how prevalence and incidence are defined and measured, particularly regarding the reference timeframes (point vs. period prevalence), data sources (community vs. hospital-based), and the population denominator used in calculations. Moreover, study designs often differ in terms of case definitions, inclusion criteria, data collection methods, and whether case validation is performed or not. This methodological variability has led to a wide range of reported estimates and limits the comparability of findings across healthcare systems and countries [18,19,20]. In Romania, there are very few epidemiological studies of the prevalence and incidence of chronic skin ulcers [21,22,23,24]. Where Romanian evidence exists, it is largely aetiology-specific and oriented toward advanced diabetic foot complications rather than nationwide surveillance of chronic ulcer incidence and prevalence across ulcer types. A nationwide cohort study quantified lower extremity amputation incidence in Romania (2015–2019), highlighting the downstream burden of advanced ulcer disease but not providing ulcer-type epidemiology at the population level [25]. At the clinical level, Romanian cohorts have proposed risk-assessment approaches for amputation in patients with diabetic foot lesions, again focusing on severe end-stage outcomes rather than national ulcer surveillance [26]. More broadly, the wound care literature increasingly supports structured, algorithm-based pathways to standardize assessment, prevention, and treatment in specific wound entities (e.g., friction injuries), underscoring the value of scalable decision-support approaches that could be adapted to chronic ulcer pathways in Romania [27].

In Romania, many patients are admitted with advanced-stage ulcers after suboptimal, often prolonged management in outpatient or home settings, including empirical self-treatment, incomplete or inappropriate antibiotic treatments, and delayed referral. These factors contribute to long, repeated hospitalizations with high resource use that highlight the preventive potential of earlier, community-based intervention.

National inpatient databases, therefore, offer several advantages for quantifying hospital-treated chronic ulcer burden. They provide broad coverage of publicly delivered inpatient care, consistently capture the most severe and resource-intensive presentations, and use standardized discharge coding and routine variables that support reproducible national estimates and comparability over time. At the same time, they do not include the large number of milder ulcers managed out of hospital care, particularly in primary care, specialist outpatient clinics, long-term care facilities, homecare, long-term care facilities or the private sector. As a result, estimates derived from inpatient data should be interpreted as describing the burden of hospital-treated chronic ulcers rather than the total population burden of chronic wounds.

Against this background, the present study provides the first nationwide assessment of chronic ulcers requiring hospitalization during 2017–2022, in Romania, based on a validated case definition applied to the public hospital inpatient database. We estimate annual prevalence, incidence, and cause-specific mortality per 100,000 adults, describe time trends before and during the COVID-19 pandemic, and examine differences by ulcer categories, sex, and age group. By restricting the cohort to patients with at least two ulcer-related hospitalizations during the study period, the analysis specifically characterizes the burden of truly chronic recurrent, clinically persistent, hard-to-heal ulcers in Romania’s public hospital system and generates national indicators to support prevention, service planning, resource allocation, quality improvement, and better hospital–community care pathways.

## 2. Materials and Methods

### 2.1. Study Design and Setting

This nationwide retrospective cohort study used the Romanian public hospital National Inpatient Database to estimate annual prevalence, incidence, and in-hospital mortality of hospital-treated chronic ulcers among adults (≥18 years) from 1 January 2017 to 31 December 2022. Case ascertainment, validation of chronicity, and outcome definitions [28] are detailed in Section 2.3, Section 2.4, Section 2.5 and Section 2.6.

### 2.2. Data Source

Anonymized data were obtained from the national inpatient database maintained by the National Institute of Public Health (Bucharest, Romania). The database had already been restricted to admissions with at least one pre-specified ulcer-related ICD-10 code and contains one record per hospitalization and includes, for each admission, a unique patient identifier, age, sex, the main discharge diagnosis (ICD-10), date of admission, length of stay (days), and discharge status (cured, improved, stationary, worse, or death). Private hospitals do not routinely report to this system; therefore, the analysis reflects the public hospital sector only. This limitation is partly mitigated by national health accounts evidence: OECD (Organisation for Economic Co-operation and Development) [29] estimates indicate that public financing covered 99% of inpatient care expenditure in Romania in 2023, suggesting that inpatient care delivered outside the publicly financed/contracted system represents a small share.

### 2.3. Case Ascertainment and Clinical Meaning of ICD-10 Codes

We used routinely collected administrative hospital discharge data and did not review individual clinical charts, so ulcer type was defined solely using the ICD-10 codes assigned. We deliberately focused on the principal discharge diagnosis because it represents the primary reason for the hospitalization and is coded more consistently for admission-driving conditions across institutions and years. In contrast, ulcer codes recorded as secondary diagnoses may reflect comorbidities, historical wounds, or ulcers present but not primarily treated during that admission; including secondary diagnoses would reduce specificity by mixing admissions driven by other acute conditions and would complicate the interpretation of prevalence and incidence as indicators of hospital-treated ulcer burden. Therefore, our estimates should be interpreted as a conservative, inpatient, public-sector measure of chronic ulcers severe enough to be coded as the principal diagnosis. Because ICD-based ulcer categories mix etiological subtypes (venous, arterial, diabetic, pressure) with anatomical-site subtypes (lower limb vs. other sites), we hereafter refer to them simply as chronic ulcer categories/types. Even within these ICD categories, recorded “chronic ulcer” diagnoses likely encompass a heterogeneous mix: long-standing chronic ulcers, newly diagnosed or short-duration ulcers; postoperative or traumatic lesions; and ulcerated tumours or inflammatory dermatoses that may be miscoded under chronic ulcer labels. This reflects the limits of routine administrative coding, where aetiology and chronicity are not always documented consistently.

### 2.4. Validation of Chronicity (≥2 Hospitalizations)

**Figure 1:**
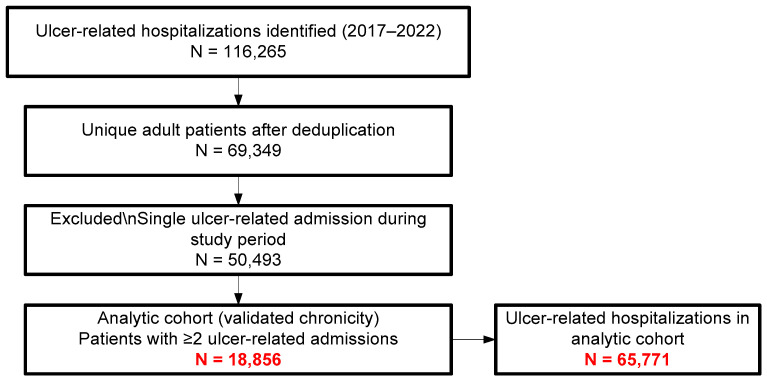
STROBE-style flow diagram of case extraction, patient deduplication, and chronicity validation (≥2 ulcer-related admissions) for the analytic cohort; red values highlight the final analytic cohort counts (patients and hospitalisations).

To increase specificity for chronic, hard-to-heal ulcers, we required evidence of recurrent hospital use, defined as ≥2 ulcer-related hospitalizations during 2017–2022 for the same unique patient identifier. Patients with a single ulcer-related admission during the study period were excluded from the analytic cohort. The extraction, deduplication, and validation steps (with corresponding counts) are summarized in Figure 1.

Patients were stratified into six ulcer categories based on the ICD-10 codes: venous ulcers (I83.x—varicose veins of lower extremities with ulceration); arterial ulcers (I70.23—atherosclerosis of arteries of extremities with ulcerations); diabetic ulcers (E1x.73—diabetes mellitus with leg ulceration of multiple causes); pressure ulcers (L89—decubitus ulceration), unclassified leg ulcers (L97—leg ulcer unclassified elsewhere); and unclassified skin ulcers (L98.4—chronic skin wound unclassified elsewhere); along with six age groups: <45, 45–54, 55–64, 65–74, 75–84, and ≥85 years.

### 2.5. Handling of Changing Diagnoses Across Admissions

For admission-level analyses, we used the ICD-10 principal diagnosis exactly as recorded at each hospitalization and did not attempt to reconcile discrepancies across admissions for the same patient.

For the age- and sex-specific distribution of ulcer type, across the whole study period, each patient in the validated cohort contributed to a single ulcer category to avoid double-counting across types. For the pooled age- and sex-specific distribution of ulcer type across 2017–2022, the validated cohort contributed to a single ulcer category to avoid double-counting across types. For patients with admissions coded under more than one ulcer category, we assigned a single “predominant” ulcer type using a two-step operational rule: (i) we selected the ulcer category most frequently recorded as the principal diagnosis across that patient’s ulcer-related admissions (modal category); (ii) if two or more categories were equally frequent, we applied a pre-specified tie-break hierarchy: L89 (pressure) → E1x.73 (diabetic) → I70.23 (arterial) → I83.x (venous) → L97 (unspecified leg) → L98.4 (unspecified skin).

This hierarchy is not intended as a universal clinical severity index (severity frameworks are etiology-specific), but as a pragmatic tie-breaker consistent with published prognostic severity patterns and with the need to prevent clinically severe phenotypes from being redistributed into non-specific residual ICD-10 categories (L97/L98.4) when coding varies across admissions. Importantly, this predominant ulcer assignment was used only for the pooled patient-level ulcer-type distribution; all admission-level analyses and annual indicators used the recorded principal ICD-10 code unchanged.

This approach was motivated by observed cross-admission coding variability, as 23.84% of patients with ≥2 admissions had more than one ulcer category recorded across hospitalizations.

For indicators that are patient-based per calendar year (prevalence, incidence and mortality), each individual was counted only once per year, at their first ulcer-related admission in that year, even if they had multiple subsequent hospitalizations or small changes in the recorded ulcer code.

### 2.6. Outcomes and Epidemiological Definitions

Annual prevalence (period prevalence) was calculated by dividing the number of individuals hospitalized for chronic ulcers (existing and new cases) in one year by the resident adult population of Romania on 1st of January of the same year and expressed per 100,000 [30,31,32]. If an individual had multiple hospitalizations within one year, they were counted only once, based on their first admission in that year.

(1) $$Prevalence=\frac{number\;of\;individuals\;hospitalised\;for\;chronic\;ulcers\;in\;one\;year}{adult\;population\;of\;that\;year,\;on\;1^{st}\;of\;January}\times 100,000$$

Annual incidence was calculated by dividing the number of new individuals hospitalized for chronic ulcers in one year by the resident adult population of Romania on 1st of January of the same year and expressed per 100,000 [30,31,32]. Individuals were considered new cases only if they had no ulcer-related admissions in any earlier study year. Because 2017 was used as the look-back year, incidence was first calculated for 2018.

(2) $$Incidence=\frac{number\;of\;new\;individuals\;hospitalised\;for\;chronic\;ulcers\;in\;one\;year}{adult\;population\;of\;that\;year,\;on\;1^{st}\;of\;January}\times 100,000$$

Annual in-hospital mortality among chronic ulcer patients (cause-specific mortality) was calculated by dividing the number of deaths occurring among the individuals hospitalized for chronic wounds in one year by the resident adult population of Romania at midpoint of the same year, on 1st of July, and expressed per 100,000 [30,31,33].

(3) $$Mortality=\frac{number\;of\;in\;hospital\;deaths\;among\;chronic\;wound\;patients\;in\;one\;year}{adult\;population\;of\;that\;year,\;on\;1^{st}\;of\;July}\times 100,000$$

From a strict epidemiological perspective, because the individuals in the numerators are also part of the corresponding population denominators over a fixed observation period, our prevalence, incidence and mortality are proportions rather than true rates [31]. Nevertheless, consistent with the chronic-wound literature, we report them as rates per 100,000 to facilitate comparability. Prevalence, incidence, and mortality were stratified by sex; age group (<45, 45–54, 55–64, 65–74, 75–84, ≥85 years); and by ulcer category (I83.x, L97, I70.23, E1x.73, L98.4, L89).

### 2.7. Statistical Analysis

Descriptive statistical analyses were performed in a Jupyter Notebook environment using Python 3.11.14, primarily with Pandas 2.3.3 library for data management and aggregation and Matplotlib 3.10.8 for plotting. Annual indicators per 100,000 adults were calculated by combining case counts from the validated cohort with official adult population denominators from the National Institute of Statistics for each calendar year. Trends were plotted by year and stratified by sex, age group, and ulcer type. Cross-tabulations of age and sex by ulcer categories were also generated. No imputation was performed. All analyses were conducted on the validated cohort. AI-assisted tools via ChatGPT (model GPT-5.2) were used only to support manuscript drafting and language editing, and to assist with assembling author-generated plots into composite figures; they were not used for data processing or statistical analysis, and all outputs and final interpretations were verified by the authors.

### 2.8. Ethical Standards

This study used an anonymized administrative database in which all personal identifiers were encoded before delivery to the authors. Access to the data was granted under a data-sharing agreement with the National Institute of Public Health, in compliance with the EU General Data Protection Regulation (GDPR) and national legislation on secondary use of health data. The protocol was approved by the Scientific Research Ethics Committee of “Lucian Blaga” University of Sibiu (approval No. 11/14 March 2025).

## 3. Results

### 3.1. Study Population, Diagnosis Validation

In total, 116,265 ulcer-related hospitalizations were identified over the six-year study period. Collapsing records by the unique patient identifier yielded 69,349 adults with at least one such hospitalization. Among these, 50,493 patients had only a single admission. As our analysis focuses on chronicity and recurrence, consistent with the predefined case definition, this subgroup was excluded from further analysis. The remaining 18,856 patients, who had two or more admissions, accounted for 65,771 hospitalizations during the study period. This subset, representing cases with repeated hospitalizations and thus a greater likelihood of a confirmed chronic condition, is considered the validated database and forms the basis of the current epidemiological analysis (Figure 1).

Across repeated admissions, we assessed consistency of ulcer type using the principal ICD-10 ulcer category recorded at each hospitalization. Among the 18,856 patients with ≥2 ulcer-related admissions, 4495 (23.84%) had more than one ulcer category recorded across admissions (inconsistent categories). Most inconsistent cases had two categories (*n* = 4234; 94.2%), followed by three categories (*n* = 245; 5.5%) and four categories (*n* = 16; 0.4%). The most frequent inconsistency was between venous ulcers (I83.x) and unspecified leg ulcers (L97), observed in 2024 patients (45.0% of inconsistent cases).

The total number of patients contributing to the annual prevalence counts (*n* = 38,099) is higher than the size of the validated cohort (*n* = 18,856) because the same patient can be hospitalized in more than one calendar year. Annual prevalence, therefore, captures both newly incident and previously hospitalized patients within each year, whereas incidence is restricted to patients whose first ulcer-related hospitalization during 2018–2022 occurred in that year.

Within the validated cohort of 18,856 patients, distinct demographic profiles were observed across ulcer aetiologies. Table 1 presents the distribution of the entire cohort by age, sex, and ulcer type. While venous ulcers showed a female predominance in older age groups, arterial and diabetic ulcers were significantly more frequent in men. This baseline demographic characterization sets the context for the subsequent prevalence and incidence analyses.

A detailed analysis of Table 1, which outlines the age- and sex-specific distribution by ulcer type, reveals distinct epidemiological profiles. Venous ulcers (I83.x) were most prominent in women aged 65–74 (*n* = 1734) and 75–84 (*n* = 1524), substantially outnumbering men in the same age brackets. In sharp contrast, arterial ulcers (I70.23) and diabetic ulcers (E1x.73) showed a strong male predominance. Men aged 55–64 and 65–74 accounted for most diabetic ulcer cases (*n* = 317 and *n* = 294, respectively), and arterial ulcers were most common in men aged 65–74 (*n* = 616). Pressure ulcers (L89), however, were more frequently recorded in elderly women, particularly in the 75–84 (*n* = 144) and ≥85 (*n* = 67) age groups, compared to men in the same categories. Note: L89—pressure ulcers; E1x.73—diabetic ulcers; I70.23—arterial ulcers; I83.x—venous ulcers; L97—unclassified leg ulcers; L98.4—chronic/unclassified skin ulcers.

### 3.2. Epidemiological Study

The prevalence of chronic wound hospitalizations in Romania varied significantly across the six-year study period. In 2017, a total of 7878 patients were hospitalized for chronic wounds, corresponding to a prevalence of 49.42 per 100,000. This figure increased in 2018 to 8901 patients (56.14/100,000), marking the highest recorded prevalence. In subsequent years, hospitalizations dropped to 8785 cases in 2019 (55.74/100,000), and in pandemic years, sharply fell to 4292 cases in 2020 (27.37/100,000) and 3525 in 2021 (22.64/100,000), the lowest point in the dataset. A partial rebound occurred in 2022, with 4718 cases (30.61/100,000) (Table 2).

Across the entire study period, venous ulcers (I83.x) remained the most prevalent subtype of chronic wounds. The number of hospitalizations peaked at 4979 cases in 2018 (31.40 per 100,000), followed by 4547 cases in 2017 (28.53), and declined sharply to 1550 cases in 2021 (9.95). Unclassified leg ulcers (L97) were the second most common, with annual case counts ranging from 1842 to 2122 and prevalence rates between 11.56 and 13.38 per 100,000, decreasing to 6.27 in 2021. Arterial ulcers (I70.23) accounted for 531 to 653 cases in the earlier years of the study (3.33–4.14 per 100,000) but declined to 360–383 cases (2.31–2.49) by 2021–2022. Other wound types, such as diabetic ulcers (E1x.73), unclassified skin ulcers (L98.4) and pressure ulcers (L89), were less frequent and showed lower prevalence levels overall. Diabetic ulcer prevalence was around 2.10 per 100,000 adults in 2017 and remained between approximately 1.5 and 1.8 per 100,000 in 2020–2022. Unclassified skin ulcers (L98.4) varied between about 2.6 and 3.4 per 100,000, with a temporary dip in 2020–2021 and an increase to 3.36 per 100,000 in 2022. Pressure ulcers (L89) were consistently the least prevalent category, with rates between 0.72 and 1.44 per 100,000 adults over the entire study period.

**Figure 2:**
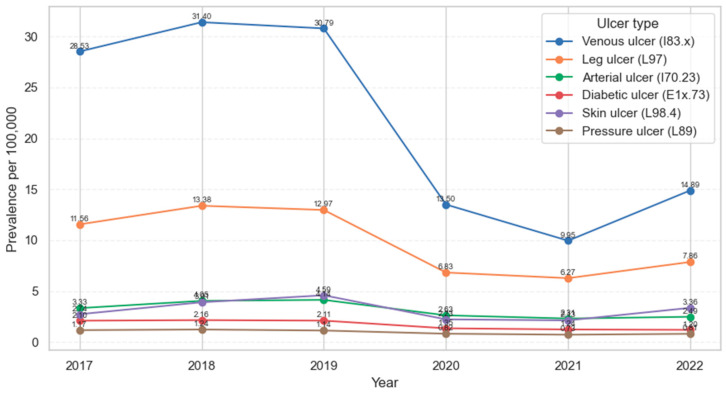
Annual prevalence (per 100,000) adults of patients hospitalized for chronic ulcers, temporal trends and differences across ulcer types, in Romania, 2017–2022.

Figure 2 displays the temporal trend in prevalence for six major ulcer types. I83.x (venous ulcers) maintained the highest prevalence throughout the study period, followed by L97 (unclassified elsewhere leg ulcers).

**Figure 3:**
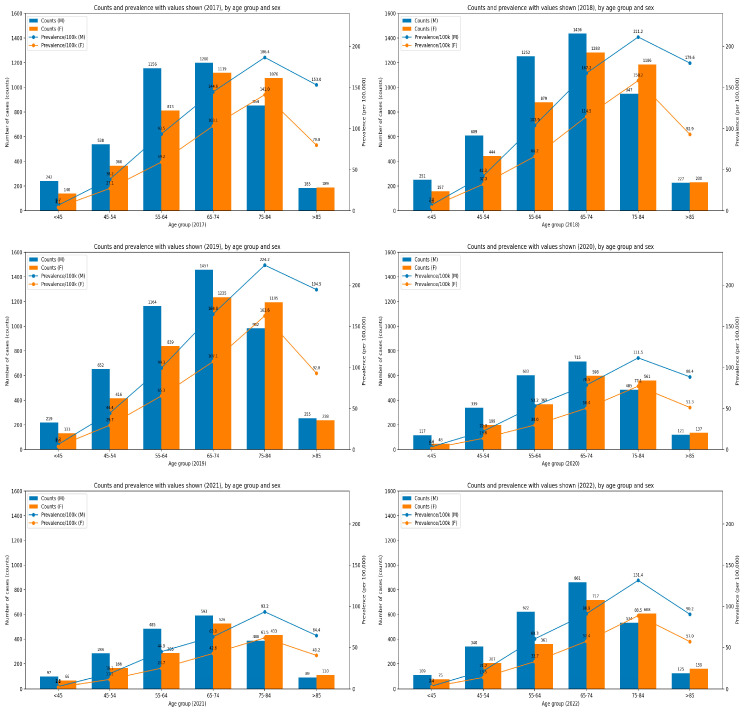
Number of cases vs. prevalence (per 100,000 adults) of patients hospitalized for chronic ulcers adults, differences across age group and sex in Romania, 2017–2022.

Age-stratified data further reveals that across all years, the highest numbers of cases were observed in the 65–74 years age group. Among men, the second-largest number of patients was seen in the 55–64-year age group, whereas among women, the second-highest number of cases occurred in those aged 75–84 years. Prevalence increased steeply with age and was consistently higher in men than in women; the highest values were observed in the 75–84-year age group for both sexes. A marked decline is visible in both counts and prevalence in 2020–2021, followed by a partial rebound in 2022 (Figure 3).

In Figure 3, bar plots show the number of cases of hospitalizations for chronic ulcers, and the overlaid lines indicate the prevalence of hospitalizations for chronic ulcers per 100,000 adults in Romania 2017–2022, stratified by age and sex (blue for males and orange for females).

**Figure 4:**
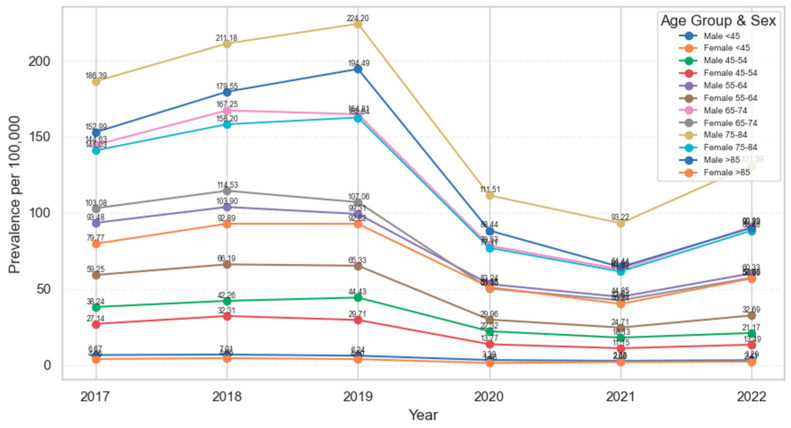
Annual prevalence (per 100,000 adults) of patients hospitalized for chronic ulcers, temporal trends and differences across age group and sex.

In Figure 4, annual prevalence rose steeply with age up to the 75–84-year group and was consistently higher in men than in women across all age groups. Prevalence peaked in 2018–2019, with the highest values observed in adults aged 75–84 years in both sexes, and somewhat lower but still elevated levels in those aged ≥ 85 years. A sharp decline was seen in 2020, followed by a further modest decrease in 2021 and only partial recovery in 2022; at every time point, female prevalence remained lower than male prevalence in the corresponding age group.

The annual incidence of chronic wound hospitalizations in Romania, calculated as newly hospitalized patients not recorded in previous study years, showed a steady decrease between 2018 and 2022 (Table 3). In 2018, the first year for incidence estimation, 4567 new patients were identified, corresponding to 28.8 per 100,000 adults. In 2019, the number of new cases decreased to 3234 (20.5/100,000). The most pronounced reduction was observed during the COVID-19 years, with 1014 new cases in 2020 (6.47/100,000) and 1058 in 2021 (6.79/100,000), representing about one quarter of the 2018 level. In 2022, incidence increased slightly to 1105 new hospitalizations (7.17/100,000), indicating only a partial return to pre-pandemic levels.

Venous ulcers (I83.x) remained the leading cause of new ulcer-related hospitalizations in all years, accounting for 2362 incident cases in 2018 (14.9/100,000) and 386 cases in 2022 (2.50/100,000). Unclassified leg ulcers (L97) ranked second, with 1029 cases (6.49/100,000) in 2018 and 250 cases (1.62/100,000) in 2022. Arterial ulcers (I70.23) showed a similar pattern, from 418 incident cases in 2018 (2.64/100,000) to 145 cases in 2022 (0.94/100,000). Diabetic ulcers (E1x.73) and unclassified skin ulcers (L98.4) followed the same pandemic-related decline. Pressure ulcers (L89), although the least frequent incident diagnosis, were recorded in 118 cases in 2018 (0.75/100,000) and decreased to 68 cases in 2022 (0.44/100,000).

Men consistently exhibited higher incidence rates than women throughout the study period. In 2018, 2411 incident cases were recorded in men (31.48/100,000) compared with 2156 in women (26.30/100,000). By 2022, incidence had fallen to 594 cases in men (7.98/100,000) and 511 in women (6.41/100,000). The highest age-specific incidence was observed in the 75–84 and ≥85 years groups in both sexes: in 2018, men aged 75–84 years reached 98.3/100,000 and women 76.9/100,000, and this age-related pattern remained present in later years, although with lower absolute case counts during the COVID-19 period.

**Figure 5:**
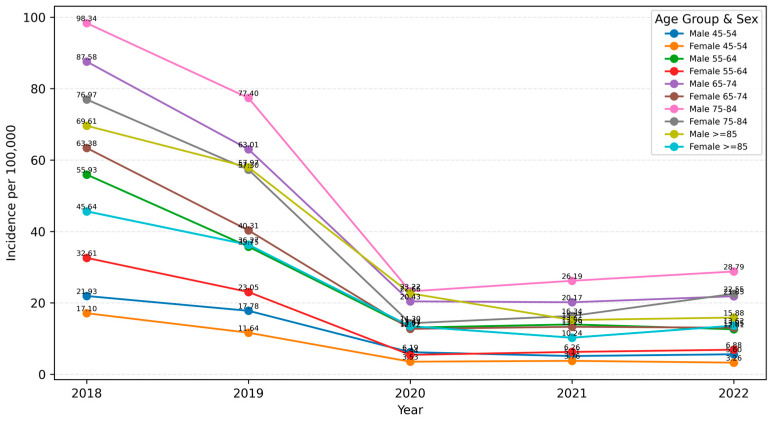
Annual incidence (per 100,000 adults) of patients hospitalized for chronic ulcers, temporal trends by age group and sex, Romania, 2018–2022.

In Figure 5, the annual incidence of chronic ulcer hospitalizations peaked in 2018 across all age groups, with the highest values observed in men aged 65–74 and 75–84 years, followed by a decline in 2019 and a marked drop in 2020 at the start of the COVID-19 pandemic. Only a modest recovery was seen in 2021–2022, and incidence in the oldest age groups remained well below pre-pandemic levels. At every time point, female incidence was lower than male incidence in the corresponding age groups.

Across 2017–2022, ulcer-attributable in-hospital mortality remained very low at the population level, ranging from 0.2155 to 0.3816 deaths per 100,000 adults. Mortality peaked in 2019 with a value of 0.3816/100,000 (60 deaths). The lowest level was 0.2155/100,000 (33 deaths) in 2022. No distinct increase in mortality was observed during 2020–2021 (36 deaths; 0.2305/100,000 in 2020 and 40 deaths; 0.2592/100,000 in 2021). Detailed age- and sex-specific mortality rates are presented in Table 4.

**Figure 6:**
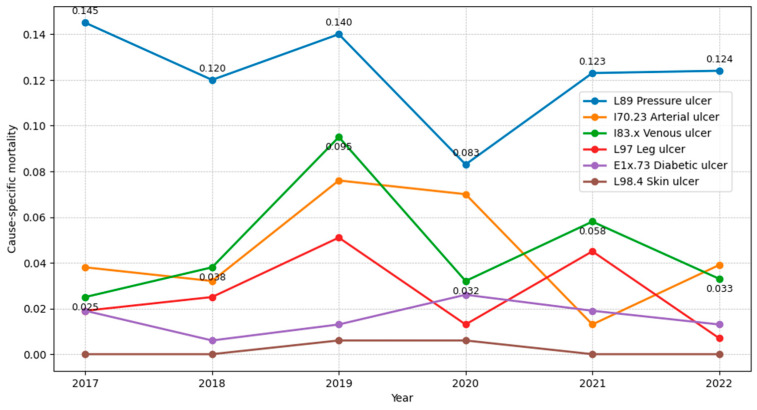
Annual mortality of patients hospitalized for chronic ulcers per 100,000 adults by ulcer type, Romania, 2017–2022.

Across all years, pressure ulcers (L89) were the dominant contributor to mortality, accounting for 36–59% of annual deaths and showing the highest mortality rates each year (e.g., 0.1447/100,000 in 2017; 0.1399/100,000 in 2019; 0.1241/100,000 in 2022). Venous ulcers (I83.x) and arterial ulcers (I70.23) were associated with intermediate mortality, with clear year-to-year variability; both peaked in 2019 (I83.x: 15 deaths; 0.0954/100,000, I70.23: 12 deaths; 0.0763/100,000) and remained substantial in 2020 for arterial ulcers (11 deaths; 0.0704/100,000). Diabetic ulcers (E1x.73) and unclassified leg ulcers (L97) were uncommon causes of death (typically 1–8 deaths/year, generally ≤ 0.0509/100,000). Unclassified skin ulcers (L98.4) were rare, with isolated deaths only in 2019–2020 (1 death/year; ~0.0064/100,000) and zero in other years (Figure 6).

Figure 6 shows annual in-hospital mortality rates per 100,000 adults for each chronic ulcer category in Romania from 2017 to 2022. Pressure ulcers (L89) consistently had the highest mortality, while venous (I83.x) and arterial ulcers (I70.23) contributed intermediate rates with a peak around 2019. Mortality from diabetic ulcers (E1x.73), unclassified leg ulcers (L97) and unclassified skin ulcers (L98.4) remained low throughout, with L98.4 near zero in most years. For readability, numeric value labels are displayed for L89 and I83.x only.

In the age–sex stratification, deaths were strongly concentrated in older adults. Across 2017–2022, there were 243 ulcer-attributable in-hospital deaths in total, with a slight female predominance (131 women, 53.9% vs. 112 men, 46.1%). By age, deaths rose steeply after 65 years: 72 deaths occurred in patients aged 65–74 years (38 women, 34 men), 85 deaths in those aged 75–84 years (50 women, 35 men), and 34 deaths among those aged ≥ 85 years (21 women, 13 men). In contrast, only 17 deaths occurred in patients < 55 years (5 women, 12 men). Overall, 191/243 deaths (78.6%) occurred at ages ≥ 65, confirming a marked age gradient, while the female excess in the oldest strata is consistent with the larger population of very old women at risk of frailty, immobility and pressure ulcer complications.

### 3.3. Statistical Precision and Robustness of Estimates

All rates were accompanied by 95% confidence intervals to document statistical precision. In high-frequency categories, such as venous ulcers (I83.x), confidence intervals were narrow, indicating low variability and reliable estimates (e.g., 2018 prevalence of I83.x: 31.40 per 100,000; 95% CI: 30.53–32.27). Even for lower-frequency outcomes, such as pressure ulcers (L89) or arterial ulcers (I70.23), CIs were wider, as expected with smaller counts, but remained informative for national-level surveillance and comparisons across years. Temporal changes observed in the study, particularly the decline during the COVID-19 period, were large in magnitude and generally exceeded what would be expected from random fluctuation alone, supporting interpretation as clinically meaningful shifts in hospital-treated burden. Age- and sex-specific patterns were also stable across years and consistent with the known epidemiology of chronic wounds, with higher rates in older adults and in men.

The use of a nationwide administrative dataset, combined with a strict case-validation rule (patients were retained only if they had at least two hospitalizations with an ulcer-related ICD-10 code during 2017–2022), provides good internal validity for the findings. This approach reduces the likelihood of including miscoded, acute, or self-limiting wounds and increases confidence that the cohort represents patients with persistent, clinically relevant ulcer disease. The observed gap between prevalence (all active cases in a year) and incidence (newly identified cases) further supports the chronic and recurrent nature of these conditions, especially in elderly groups.

Taken together, these elements indicate that the results are statistically robust and suitable to inform national-level monitoring of chronic-wound hospitalizations in Romania and to support planning of preventive and wound care services. These indicators can be integrated into the broader framework of hospital performance metrics and contracting already used in Romania, where performance indicators play a key role in financing surgical services [34].

## 4. Discussion

### 4.1. Key Results

This study provides the first national description of hospitalized chronic ulcers in Romania over a six-year period (2017–2022), using a validated cohort that included only patients with at least two ulcer-related hospitalizations. This approach reduced potential misclassification due to coding errors and improved the specificity for chronic wounds compared with single-episode case definitions.

Across all years, venous ulcers (I83.x) were the most frequently recorded diagnosis, followed by unclassified leg ulcers (L97) and arterial ulcers (I70.23). When all validated cases were considered, the highest overall burden was observed in pre-pandemic years, with hospital prevalence peaking in 2018 at 56.14 per 100,000 adults and incidence (newly identified patients with no prior ulcer-related admission in earlier years), reaching 28.80 per 100,000 in 2018, the first year in which incidence could be estimated. Starting in 2020, both prevalence and incidence dropped sharply and recovered only partially in 2022, indicating a pandemic-related disruption of access to inpatient care rather than a true decline in chronic ulcer occurrence.

Age- and sex-stratified annual analyses revealed a highly consistent pattern. In absolute numbers, most hospitalized chronic ulcer patients were concentrated in the 65–74 and 75–84 age groups, whereas rate-based measures (prevalence and incidence) increased steeply with age and were highest in the 75–84 and ≥85 strata. Across age groups, men generally exhibited higher hospital prevalence and incidence than women, indicating an overall male excess in ulcer-related hospital burden. However, sex differences in mortality were less uniform and were strongly shaped by the concentration of deaths at older ages.

Stratifying patient counts by ulcer type, age group, and sex (Table 1) clarifies the pattern underlying the overall male predominance. In the 55–64 age group, men outnumbered women by more than fourfold for both diabetic ulcers (E1x.73) (M/F 4.06) and arterial ulcers (I70.23) (M/F 4.71). In 65–74, the male excess remained marked (M/F 2.45 for diabetic ulcers and 2.92 for arterial ulcers). By contrast, sex differences for venous ulcers were modest in 55–64 (M/F 1.13) and reversed in older age groups, with higher counts in women in 65–74 (M/F 0.79) and 75–84 (M/F 0.69). This pattern is consistent with established literature linking arterial and diabetic ulceration to higher rates of smoking, systemic atherosclerosis, and peripheral arterial disease in males. Conversely, pressure ulcer (L89) case counts were higher in women aged ≥ 65 years (65–74: 117 vs. 102; 75–84: 144 vs. 79; ≥85: 67 vs. 29). This pattern is explained by demography and care pathways: there are more women alive at older ages, so the pool of very old and frail individuals is disproportionately female. The age–sex profiles by ulcer type shown in Table 1 indicate that the overall male predominance is not confined to a single diagnostic category: men show higher and earlier age-specific burdens for diabetic and arterial ulcers, while sex differences for venous ulcers are smaller and become female-predominant in older age groups. Pressure ulcers (L89) represent a notable exception, with higher counts among women aged ≥ 65.

In our cohort, pressure ulcers (L89) carried the highest cause-specific in-hospital mortality per 100,000 adults, despite being the least prevalent ulcer subtype. Across 2017–2022, mortality associated with L89 remained consistently higher than that for other ulcer types and accounted for nearly half of all ulcer-attributed deaths. Venous (I83.x) and arterial ulcers (I70.23) showed intermediate mortality rates, whereas unclassified leg ulcers (L97), diabetic ulcers (E1x.73) and unclassified skin ulcers (L98.4) were associated with very low mortality and contributed only a small fraction of deaths over the study period. This pattern indicates that immobility-related pressure damage in frail patients remains the most lethal chronic ulcer presentation once patients are hospitalized, even though such ulcers represent only a minority of all chronic ulcer cases.

**Figure 7:**
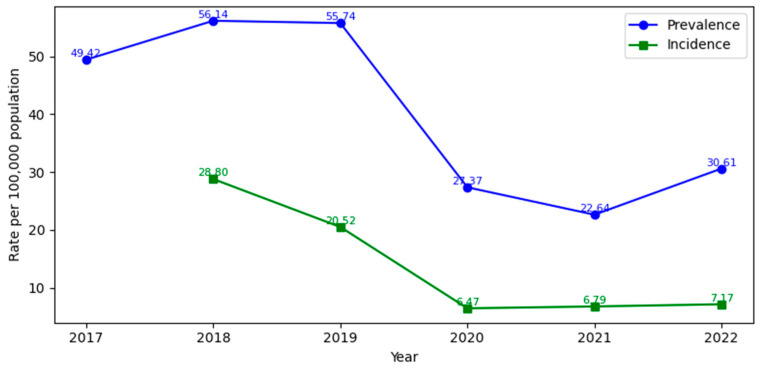
Annual prevalence (2017–2022) and incidence (2018–2022) of patients hospitalized for chronic ulcers, per 100,000 adults, in Romania.

As shown in Figure 7, annual prevalence was consistently higher than the annual incidence of patients hospitalized for chronic ulcers in every study year. This indicates that substantially more patients remain in the hospital care pathway than enter it as new cases. The persistent gap between these indicators is expected for chronic, slow-healing, or recurrent ulcers and supports the interpretation that chronic wound care in Romania deals with hard-to-manage cases in older patients who require repeated hospitalizations. In this context, annual prevalence better captures the ongoing burden of chronicity and recurrent admissions, whereas annual incidence provides a conservative estimate (lower bound) of newly hospitalized chronic ulcer patients within each year.

### 4.2. Interpretation

The epidemiological pattern identified in this study is broadly consistent with reports from other European settings, where chronic, hospital-treated ulcers primarily affect older adults and venous leg ulcers are the predominant category. The concentration of patients in the 65+ age groups and the male predominance we observed mirror these findings and support the external validity of our data.

Pressure ulcers (L89) carried the highest cause-specific in-hospital mortality per 100,000 adults, despite being less frequent than venous and unclassified leg ulcers. This pattern is clinically plausible: patients hospitalized for L89 as the principal diagnosis are typically very frail and immobilized, often with multiple comorbidities and limited life expectancy, so that pressure damage becomes a marker of advanced dependency and end-of-life care.

By contrast, patients hospitalized with diabetic foot ulcers (E1x.73) generally remain ambulant and are hospitalized for limb-salvage procedures, infection control or debridement. These ulcers generate substantial morbidity, recurrent admissions and a high risk of amputation, but in-hospital death during a given admission is relatively uncommon in our data. It is important to emphasise that the low number of deaths coded under E1x.73 in our dataset should not be interpreted as evidence that diabetic foot ulcers are benign. In routine ICD-10 coding, the fatal event in such patients is frequently attributed to cardiovascular disease, sepsis or multi-organ failure, while the ulcer is recorded as a secondary diagnosis and is therefore not captured in our cause-specific mortality estimates. Our findings thus reflect differences in coding practices and clinical contexts between ulcer types, rather than fundamentally different prognoses for the underlying diseases.

A notable finding is the high proportion of ulcers coded as L97 (“ulcer of lower limb, not elsewhere classified”). L97 is an anatomical (lower-limb) category that does not specify aetiology; in practice, it can therefore group together venous, arterial, diabetic and other leg ulcers for which no explicit cause-specific code was assigned. L98.4 (“chronic ulcer of skin, not elsewhere classified”) was less frequent but represents a similar lack of specificity for ulcers at other anatomical sites, often reflecting ulcers related to malignancy, autoimmune disease or other dermatological conditions that are not captured by site-specific or cause-specific codes. In routine hospital coding, both L97 and L98.4 tend to be selected when clinical documentation does not support a more specific diagnosis, when several causes coexist, or when the focus of care lies elsewhere. Their prominence in our data, therefore, reflects limitations of routine documentation and coding rather than distinct clinical entities. Improving coding specificity through clearer guidance and training would allow future surveillance to distinguish more precisely between venous, arterial, diabetic, pressure and other ulcer types and to better target preventive strategies.

The abrupt fall in both incidence and prevalence in 2020–2021, visible in Figure 7, is unlikely to represent a true short-term epidemiological decrease in chronic ulcer occurrence. Instead, it most plausibly reflects a pandemic-related reduction in hospital-treated capture, driven by a combination of changes in healthcare-seeking behavior (delayed presentation and avoidance of hospitals); system-level constraints on non-COVID admissions (reallocation of beds and staff, reduced elective/chronic-care capacity); and partial shifts toward outpatient, community or remote wound management that are not observable in a public inpatient database [35]. In our validated cohort, annual prevalence decreased from 55.74/100,000 in 2019 to 27.37/100,000 in 2020 and 22.64/100,000 in 2021, with only partial recovery in 2022 (30.61/100,000); incidence showed a comparable drop from 20.5/100,000 in 2019 to 6.47/100,000 in 2020 and 6.79/100,000 in 2021, with modest rebound in 2022 (7.17/100,000). National reports on Romania’s hospital activity during COVID-19 describe a sharp decline in hospital discharge rates and disrupted service provision linked to reduced capacity and changes in patient healthcare-seeking behavior, supporting interpretation as a service-availability/utilization effect rather than a true decrease in underlying disease burden. [29,35]

The persistent excess of prevalence over incidence throughout 2018–2022 confirms that chronic ulcers in Romania behave as long-lasting, recurrent conditions that accumulate over time in the hospital system. This accumulation is consistent with the age–sex patterns observed in our cohort and underscores the need for long-term, multidisciplinary management strategies focused on prevention, early detection and sustained care in older adults.

From a methodological perspective, our analytic choices follow the recommendations made by Probst and colleagues, who emphasized the need for clear case definitions, explicit population denominators and standardized observation periods to enable valid comparisons across populations [18,36]. By using ICD-10 groupings, census-based adult population denominators and a “more than 2 hospitalisations” rule to validate chronicity, this study provides a transparent framework that can serve as a national reference model for future chronic wound epidemiology in Romania.

### 4.3. Clinical Implications

Our results suggest that the inpatient burden is driven mainly by older adults (≥65 years), who account for most admissions and nearly all ulcer-attributable deaths, while the small but persistent mortality observed in this cohort is driven largely by pressure ulcer presentations (L89), which contribute disproportionately to in-hospital mortality once patients are admitted. Men appear to populate hospitals at younger ages, indicating that preventive health system interventions should begin earlier with a focus on modifiable vascular–metabolic risks, including smoking, alcohol consumption, obesity, and stress.

Before the first ulcer-related hospitalization, early recognition of neuropathy and peripheral arterial disease, together with prompt outpatient assessment of new tissue loss, is essential to prevent progression. For older and frail patients, who are disproportionately female at the oldest ages, priorities shift toward mobility preservation, adequate protein intake and hydration, and close monitoring for early skin breakdown.

During hospitalization, pressure ulcer prevention and early triage should be standardized at admission using structured risk assessment (e.g., Braden scale) [37], enforced repositioning/offloading, moisture and continence management, and nutrition/hydration support.

After discharge, avoidable readmissions can be reduced by ensuring dressing and offloading supplies, arranging follow-up within 7–14 days when feasible, mobilizing community or home nursing when available, and providing clear written self-care guidance with red-flag symptoms that require urgent reassessment.

The temporary decline in admissions during the COVID-19 period in our results implies that service disruptions can quickly translate into missed opportunities for prevention and timely treatment, underscoring the need for resilient care pathways during health-system shocks.

Formally integrating these steps into outpatient, primary care and post-discharge pathways would target exactly the subgroups that, in our data, concentrated the highest demand for inpatient care.

### 4.4. Limitations

This study has several limitations inherent to the use of administrative hospital data. First, we captured only hospitalized cases from the public sector, which likely underestimates the true burden of chronic wounds managed in outpatient, community or long-term care settings and in private hospitals. However, OECD estimates indicate that public financing covered 99% of inpatient care expenditure in Romania in 2023, suggesting that the non-captured purely private inpatient segment is small and that the direction of bias is toward underestimation rather than distortion of time trends [29].

Second, diagnostic accuracy depends on the quality and consistency of ICD-10 coding in the administrative dataset. The high use of site-defined but etiologically non-specific codes, particularly L97 (ulcer of lower limb, not elsewhere classified) and, to a lesser extent, L98.4 (chronic ulcer of skin, not elsewhere classified), suggests that the underlying cause (venous, arterial, diabetic, pressure or other) is frequently not recorded as a cause-specific code. In routine hospital coding, these residual categories may be used when clinical documentation is incomplete, when multiple aetiologies coexist, or when ulcer aetiology is not the main focus of care, and coding choices may also be shaped by local administrative and reimbursement practices. As a result, some ulcers that are venous, arterial or diabetic in origin may appear in our data under these non-specific categories. To reduce misclassification, we restricted inclusion to primary discharge diagnoses and required ≥ 2 ulcer-related hospitalizations during the study period; however, residual coding inaccuracy cannot be excluded.

Third, our incidence estimates assume near-complete capture of prevalent cases in the baseline year (2017). Incident cases identified in 2018 may therefore include a small proportion of patients whose ulcers pre-dated the observation window but were first recorded only in that year, leading to a slight overestimation of incidence in the first follow-up year.

Fourth, by requiring at least two ulcer-related admissions during 2017–2022, we intentionally excluded 50,493 patients (72.8% of all adults with at least one ulcer ICD-10 code) who had only a single recorded hospitalization. This rule was chosen a priori to increase specificity for chronic, hard-to-heal ulcers and to limit the impact of miscoding or upcoding, but it introduces selection toward patients with recurrent or prolonged hospital use and therefore underestimates the total number of individuals affected by chronic ulcers. The single-admission group is likely heterogeneous and may include milder ulcers that healed after one hospital episode; very severe ulcers in frail patients who died during or shortly after their first admission; patients whose subsequent ulcer care occurred entirely in outpatient or long-term-care settings; and patients whose additional ulcer-related hospitalizations fell outside the 2017–2022 observation window. Conducting a head-to-head analysis of the validated database (≥2 ulcer-related admissions) versus the non-validated database (single admission), as in Heyer et al., was beyond our predefined scope but remains a valuable direction for future research [14].

Within the validated cohort of 18,856 patients, our definition of chronicity was based on repeated hospital use rather than continuous follow-up of a single lesion. We could not distinguish between a single non-healing ulcer that required several closely spaced admissions and separate ulcer episodes occurring years apart (for example, an admission in 2017 and another in 2022). In addition, some patients had admissions coded under different ulcer categories over time. For descriptive age- and sex-specific analyses, we therefore assigned each patient to one predominant ulcer category using a modal-category rule with a pre-specified severity-based tie-break hierarchy of ICD-10 codes, which simplifies interpretation but does not fully capture the complexity of mixed-aetiology or changing ulcers.

Finally, our prevalence, incidence and mortality indicators only describe patients with recurrent ulcer-related hospitalizations in the public hospital system. We captured deaths that occurred during an ulcer-coded hospitalization, but deaths after discharge or during admissions with another principal diagnosis, where the ulcer was recorded only as a secondary diagnosis, were not included and may lead to underestimation of the overall mortality burden of chronic ulcers.

### 4.5. Generalizability

Although the analysis was limited to public hospital data, its nationwide coverage, six-year observation period and uniform case-definition strategy support the generalizability of the findings to hospitalized chronic ulcer patients in Romania. The epidemiological patterns we observed, older age, male predominance, venous and unclassified leg ulcers as leading diagnoses, and the pandemic-related dip in 2020–2021, are in line with reports from other European settings, which further suggests external validity of the estimates. However, extrapolation to outpatient, homecare or institutionalized populations should be made with caution, as these groups are likely to include less severe ulcers and different care pathways than those captured in inpatient data.

In conclusion, this study provides robust, nationally representative evidence on the burden and profile of chronic ulcers requiring hospitalization in Romania and highlights the need for prevention, earlier identification and better coordination between hospital and community services to reduce avoidable admissions and improve outcomes in this vulnerable population.

## 5. Conclusions

Using a nationwide public hospital inpatient database and a validated chronicity rule, we provide the first standardized inpatient indicators of hospital-treated chronic ulcer burden in Romania (prevalence, incidence, and mortality) across major ICD-10 ulcer categories. The results highlight a substantial burden concentrated in men and older adults and demonstrate pronounced sensitivity of hospital-treated indicators to healthcare access constraints during 2020–2021. These findings can inform prevention and service planning by prioritizing early detection and risk reduction in high-risk groups, strengthening integrated hospital–community wound care pathways, and supporting resource allocation for outpatient capacity to reduce avoidable admissions and complications.

Future research should integrate outpatient and inpatient data to capture the full continuum of wound care, clinically validate ICD-10 ulcer coding against chart review and clinical severity measures, and assess regional and socioeconomic gradients to guide targeted interventions. Evaluations of preventive strategies (e.g., pressure-injury prevention in institutional care, diabetic foot screening and multidisciplinary management, and earlier referral pathways) are also needed to quantify impact on admissions, amputations, and mortality.

## Figures and Tables

**Table 1:** Age- and sex-specific distribution by ulcer type of 18,856 patients with two or more hospitalizations for chronic ulcers in Romania, 2017–2022.

Age Group	Sex	Ulcer Category/Number (Percent)					
		L89	E1x.73	I70.23	I83.x	L97	L98.4
<45	men	58 (6.85%)	26 (2.18%)	14 (0.66%)	283 (2.76%)	113 (3.41%)	74 (6.68%)
	women	23 (2.72%)	6 (0.50%)	8 (0.37%)	165 (1.61%)	64 (1.93%)	93 (8.40%)
45–54	men	51 (6.02%)	139 (11.68%)	117 (5.48%)	731 (7.12%)	237 (7.15%)	104 (9.39%)
	women	26 (3.07%)	17 (1.43%)	25 (1.17%)	587 (5.72%)	174 (5.25%)	93 (8.40%)
55–64	men	95 (11.22%)	317 (26.64%)	419 (19.63%)	1238 (12.06%)	466 (14.05%)	143 (12.92%)
	women	56 (6.61%)	78 (6.55%)	89 (4.17%)	1095 (10.67%)	315 (9.50%)	124 (11.20%)
65–74	men	102 (12.04%)	294 (24.71%)	616 (28.87%)	1367 (13.32%)	554 (16.71%)	159 (14.36%)
	women	117 (13.81%)	120 (10.08%)	211 (9.89%)	1734 (16.90%)	461 (13.90%)	129 (11.65%)
75–84	men	79 (9.33%)	102 (8.57%)	335 (15.70%)	1045 (10.18%)	326 (9.83%)	74 (6.68%)
	women	144 (17.00%)	69 (5.80%)	192 (9.00%)	1524 (14.85%)	446 (13.45%)	73 (6.59%)
≥85	men	29 (3.42%)	14 (1.18%)	55 (2.58%)	235 (2.29%)	67 (2.02%)	23 (2.08%)
	women	67 (7.91%)	8 (0.67%)	53 (2.48%)	258 (2.51%)	93 (2.80%)	18 (1.63%)
Total 18,856		847	1190	2134	10,262	3316	1107

Note: I83.x—venous ulcers; L97—unclassified leg ulcers; I70.23—arterial ulcers; E1x.73—diabetic ulcers; L89—pressure ulcers; L98.4—chronic/unclassified skin ulcers.

**Table 2:** Annual number, percentage, prevalence (per 100,000 adults) and 95% confidence intervals of patients hospitalized for chronic ulcers, by ulcer type and total, in Romania, 2017–2022.

Year/Population		I83.x	L97	I70.23	E1x.73	L98.4	L89	Total
2017 / 15,939,431	n	4547	1842	531	335	436	187	7878
	%	57.72	23.38	6.74	4.25	5.53	2.37	100
	prevalence	28.53	11.56	3.33	2.1	2.74	1.17	49.42
	95% CI	27.7–29.36	11.03–12.08	3.05–3.61	1.88–2.33	2.48–2.99	1.01–1.34	48.33–50.52
2018 / 15,856,123	n	4979	2122	642	342	620	196	8901
	%	55.94	23.84	7.21	3.84	6.97	2.2	100
	prevalence	31.4	13.38	4.05	2.16	3.91	1.24	56.14
	95% CI	30.53–32.27	12.81–13.95	3.74–4.36	1.93–2.39	3.6–4.22	1.06–1.41	54.97–57.3
2019 / 15,759,889	n	4853	2044	653	332	724	179	8785
	%	55.24	23.27	7.43	3.78	8.24	2.04	100
	prevalence	30.79	12.97	4.14	2.11	4.59	1.14	55.74
	95% CI	29.93–31.66	12.41–13.53	3.83–4.46	1.88–2.33	4.26–4.93	0.97–1.3	54.58–56.91
2020 / 15,683,675	n	2118	1071	412	212	350	129	4292
	%	49.35	24.95	9.6	4.94	8.15	3.01	100
	prevalence	13.5	6.83	2.63	1.35	2.23	0.82	27.37
	95% CI	12.93–14.08	6.42–7.24	2.37–2.88	1.17–1.53	2.0–2.47	0.68–0.96	26.55–28.18
2021 / 15,571,548	n	1550	977	360	193	332	113	3525
	%	43.97	27.72	10.21	5.48	9.42	3.21	100
	prevalence	9.95	6.27	2.31	1.24	2.13	0.73	22.64
	95% CI	9.46–10.45	5.88–6.67	2.07–2.55	1.06–1.41	1.9–2.36	0.59–0.86	21.89–23.38
2022 / 15,412,147	n	2295	1212	383	185	518	125	4718
	%	48.64	25.69	8.12	3.92	10.98	2.65	100
	prevalence	14.89	7.86	2.49	1.2	3.36	0.81	30.61
	95% CI	14.28–15.5	7.42–8.31	2.24–2.73	1.03–1.37	3.07–3.65	0.67–0.95	29.74–31.49

Note: I83.x—venous ulcers; L97—unclassified leg ulcers; I70.23—arterial ulcers; E1x.73—diabetic ulcers; L89—pressure ulcers; L98.4—chronic/unclassified skin ulcers; population—the country’s adult population on 1 January of that year.

**Table 3:** Annual number, percentage, incidence (per 100,000 adults) and 95% confidence intervals of patients hospitalized for chronic ulcers, by ulcer type and total, in Romania, 2018–2022.

Year/Population		I83.x	L97	I70.23	E1x.73	L98.4	L89	Total
2018/	n	2362	1029	418	223	396	139	4567
15,856,123	%	51.72	22.53	9.15	4.88	8.67	3.04	100
	incidence	14.9	6.49	2.64	1.41	2.5	0.88	28.8
	95% CI	14.3–15.5	6.09–6.89	2.38–2.89	1.22–1.59	2.25–2.74	0.73–1.02	27.97–29.64
2019/	n	1517	740	357	174	328	118	3234
15,759,889	%	46.91	22.88	11.04	5.38	10.14	3.65	100
	incidence	9.63	4.7	2.27	1.1	2.08	0.75	20.52
	95% CI	9.14–10.11	4.36–5.03	2.03–2.5	0.94–1.27	1.86–2.31	0.61–0.88	19.81–21.23
2020/	n	348	240	180	79	91	76	1014
15,683,675	%	34.32	23.67	17.75	7.79	8.97	7.5	100
	incidence	2.22	1.53	1.15	0.5	0.58	0.48	6.47
	95% CI	1.99–2.45	1.34–1.72	0.98–1.32	0.39–0.61	0.46–0.7	0.38–0.59	6.07–6.86
2021/	n	343	272	173	72	124	74	1058
15,571,548	%	32.42	25.71	16.35	6.81	11.72	6.99	100
	incidence	2.2	1.75	1.11	0.46	0.8	0.48	6.79
	95% CI	1.97–2.44	1.54–1.95	0.95–1.28	0.36–0.57	0.66–0.94	0.37–0.58	6.39–7.2
2022/	n	386	250	145	69	187	68	1105
15,412,147	%	34.93	22.62	13.12	6.24	16.92	6.15	100
	incidence	2.5	1.62	0.94	0.45	1.21	0.44	7.17
	95% CI	2.25–2.75	1.42–1.82	0.79–1.09	0.34–0.55	1.04–1.39	0.34–0.55	6.75–7.59

Note: I83.x—venous ulcers; L97—unclassified leg ulcers; I70.23—arterial ulcers; E1x.73—diabetic ulcers; L98.4—chronic skin ulcer, other/unspecified; L89—pressure ulcers; population—adult population of Romania on 1 January of the corresponding year.

**Table 4:** Annual number, percentage, mortality (per 100,000 adults) and 95% confidence intervals of patients hospitalized for chronic ulcers, by ulcer type and total Romania, 2017–2022.

Year/Population		I83.x	L97	I70.23	E1x.73	L98.4	L89	Total
2017/ 15,896,137	n	4	3	6	3	0	23	39
	%	10.26	7.69	15.38	7.69	0.00	58.97	100
	mortality	0.025	0.019	0.038	0.019	0.000	0.145	0.245
	95% CI	0.007–0.064	0.004–0.055	0.014–0.082	0.004–0.055	0.000–0.023	0.092–0.217	0.174–0.335
2018/ 15,803,757	n	6.00	4.00	5.00	1.00	0.00	19.00	35
	%	17.14	11.43	14.29	2.86	0.00	54.29	100
	mortality	0.038	0.025	0.032	0.006	0.000	0.120	0.221
	95% CI	0.014–0.083	0.007–0.065	0.010–0.074	0.000–0.035	0.000–0.023	0.072–0.188	0.154–0.308
2019/ 15,721,399	n	15.00	8.00	12.00	2.00	1.00	22.00	60
	%	25.00	13.33	20.00	3.33	1.67	36.67	100
	mortality	0.095	0.051	0.076	0.013	0.006	0.140	0.382
	95% CI	0.053–0.157	0.022–0.100	0.039–0.133	0.002–0.046	0.000–0.035	0.088–0.212	0.291–0.491
2020/ 15,617,687	n	5.00	2.00	11.00	4.00	1.00	13.00	36
	%	13.89	5.56	30.56	11.11	2.78	36.11	100
	mortality	0.032	0.013	0.070	0.026	0.006	0.083	0.231
	95% CI	0.010–0.075	0.002–0.046	0.035–0.126	0.007–0.066	0.000–0.036	0.044–0.142	0.161–0.319
2021/ 15,429,205	n	9.00	7.00	2.00	3.00	0.00	19.00	40
	%	22.50	17.50	5.00	7.50	0.00	47.50	100
	mortality	0.058	0.045	0.013	0.019	0.000	0.123	0.259
	95% CI	0.027–0.111	0.018–0.093	0.002–0.047	0.004–0.057	0.000–0.024	0.074–0.192	0.185–0.353
2022/ 15,309,736	n	5.00	1.00	6.00	2.00	0.00	19.00	33
	%	15.15	3.03	18.18	6.06	0.00	57.58	100
	mortality	0.033	0.007	0.039	0.013	0.000	0.124	0.216
	95% CI	0.011–0.07	0.000–0.036	0.014–0.085	0.002–0.047	0.000–0.024	0.075–0.194	0.148–0.303

Note: I83.x—venous ulcers; L97—unclassified leg ulcers; I70.23—arterial ulcers; E1x.73—diabetic ulcers; L89—pressure ulcers; L98.4—chronic/unclassified skin ulcers; population—the country’s adult population on 1 July of that year.

## Data Availability

The dataset is available upon request from the authors. The raw data supporting the conclusions of this article will be made available by the authors on request.
